# Network Properties of Complex Human Disease Genes Identified through Genome-Wide Association Studies

**DOI:** 10.1371/journal.pone.0008090

**Published:** 2009-11-30

**Authors:** Fredrik Barrenas, Sreenivas Chavali, Petter Holme, Reza Mobini, Mikael Benson

**Affiliations:** 1 The Unit for Clinical Systems Biology, University of Gothenburg, Gothenburg, Sweden; 2 Department of Physics, Umeå University, Umeå, Sweden; 3 Department of Energy Science, Sungkyunkwan University, Suwon, Korea; Aarhus University, Denmark

## Abstract

**Background:**

Previous studies of network properties of human disease genes have mainly focused on monogenic diseases or cancers and have suffered from discovery bias. Here we investigated the network properties of complex disease genes identified by genome-wide association studies (GWAs), thereby eliminating discovery bias.

**Principal findings:**

We derived a network of complex diseases (n = 54) and complex disease genes (n = 349) to explore the shared genetic architecture of complex diseases. We evaluated the centrality measures of complex disease genes in comparison with essential and monogenic disease genes in the human interactome. The complex disease network showed that diseases belonging to the same disease class do not always share common disease genes. A possible explanation could be that the variants with higher minor allele frequency and larger effect size identified using GWAs constitute disjoint parts of the allelic spectra of similar complex diseases. The complex disease gene network showed high modularity with the size of the largest component being smaller than expected from a randomized null-model. This is consistent with limited sharing of genes between diseases. Complex disease genes are less central than the essential and monogenic disease genes in the human interactome. Genes associated with the same disease, compared to genes associated with different diseases, more often tend to share a protein-protein interaction and a Gene Ontology Biological Process.

**Conclusions:**

This indicates that network neighbors of known disease genes form an important class of candidates for identifying novel genes for the same disease.

## Introduction

Systems Biology based approaches of studying human genetic diseases have brought in a shift in the paradigm of elucidating disease mechanisms from analyzing the effects of single genes to understanding the effect of molecular interaction networks. Such networks have been exploited to find novel candidate genes, based on the assumption that neighbors of a disease-causing gene in a network are more likely to cause either the same or a similar disease [1]–[14]. Initial studies investigating the network properties of human disease genes were based on cancers and revealed that up-regulated genes in cancerous tissues were central in the interactome and highly connected (often referred to as hubs) [1], [2]. A subsequent study based on the human disease network and disease gene network derived from the Online Mendelian Inheritance in Man (OMIM) demonstrated that the products of disease genes tended (i) to have more interactions with each other than with non-disease genes, (ii) to be expressed in the same tissues and (iii) to share Gene Ontology (GO) terms [8]. Contradicting earlier reports, this latter study demonstrated that the non-essential human disease genes showed no tendency to encode hubs in the human interactome. A more recent report that evaluated the network properties of disease genes showed that genes with intermediate degrees (numbers of neighbors) were more likely to harbor germ-line disease mutations [12]. However, interpretation of this dataset might not be applicable to complex disease genes since 97% of the disease genes were monogenic. Despite this reservation, both the latter studies found a functional clustering of disease genes. Another concern is that the above studies could be confounded by discovery bias, in other words these disease genes were identified based on previous knowledge. By contrast, Genome Wide Association studies (GWAs) do not suffer from such bias [15].

In this study, we have derived networks of complex diseases and complex disease genes to explore the shared genetic architecture of complex diseases studied using GWAs. Further, we have evaluated the topological and functional properties of complex disease genes in the human interactome by comparing them with essential, monogenic and non-disease genes. We observed that diseases belonging to the same disease class do not always show a tendency to share common disease genes; the complex disease gene network shows high modularity comparable to that of the human interactome; complex disease genes associated with same disease more often tend to share a protein-protein interaction (PPI) and GO biological process in comparison to the genes associated with different diseases. We demonstrate that complex disease genes are less central to the essential and monogenic disease genes in the molecular interaction network. Our results support the assumption that novel candidate genes might be identified among the network neighbors of known complex disease genes.

## Methods

### Data Sources

We obtained the list of complex human disease associated genes identified by GWAs (Table S1) from ‘A catalog of published genome-wide association studies’ (retrieved on March 23, 2009) [16]. The complex disease genes catalogued in this database have SNPs published with a p-value less than 1E-5. The complex disease genes dataset contained 349 genes that were implicated in 54 complex diseases. Disease classes of these diseases were identified using MeSH (Medical Subject Headings) terms. We retrieved a list of monogenic disease genes (n = 738) from the compendium compiled by Jimenez-Sanchez et al [17]. Essential genes were defined as previously described [8]. Briefly, a list of human orthologs of mouse genes that resulted in a lethal phenotype in embryonic and postnatal stages upon knockout was obtained from the Mouse Genome database [18]. Next, complex and monogenic disease genes were removed from that list resulting in 1986 essential genes. To construct a human interactome, we obtained 35,021 protein-protein interactions (PPIs) pertaining to 9462 proteins from the Human Protein Reference Database (HPRD) database (release 7) [19], as it is known to be one of the most reliable databases for PPI data [20]. Non-essential genes without any disease associations and with interactions in HPRD were considered as non-disease genes (n = 6659).

### Construction of Complex Disease and Complex Disease Gene Networks

We constructed two separate networks from the compendium of human complex diseases and their associated genes. In the complex disease network (CDN), the nodes of the network are diseases and two diseases were connected if they shared an associated gene; in the complex disease gene network (CGN) nodes are genes and a link represents two genes associated with the same disease. To investigate how the topology of CGN differed from random null model networks we randomly rewired the links between genes 1000 times, while keeping degrees constant. P-values for assortativity and modularity of networks have been estimated as the ratio of randomized models with a higher value of the corresponding topological parameter than that of the real networks. To investigate if the CGN shared topological features with the interactome we applied the same method to the interactome data. (A detailed description is given in Supplementary Material S1.) Such rewiring methods need large networks to be interpretable. Due to its small number of edges, we avoided these methods for analyzing the CDN.

### Topological and Functional Properties of Complex Disease Genes in Human Interactome

In many types of networks, including gene networks, one can assume that the importance of a node (for example the likelihood of causing a disease) is correlated with centrality. There is, however, not only one way of measuring network centrality; rather there are different types of measures trying to capture different aspects of the concept. We measure three different centrality quantities — *degree*, *closeness* and *eccentricity*. Closeness is defined as the reciprocal average distance (number of links in the shortest path) to every other node — a node with high closeness is thus, on average, close in graph distance to the other nodes. The eccentricity of a node is the distance to the farthest reachable other node in a network, thus focusing on a maximal property where closeness focus on an average. We compare these topological measures between different classes of genes by Mann–Whitney U tests.

To determine functional similarity among genes causing the same disease compared to genes causing different diseases, we used the Biological Process category of Gene Ontology classification. We also determined the enriched terms among our complex disease genes compared to all genes listed in Entrez database, using the Bioconductor package TopGO [21].

## Results

### Complex Diseases Network (CDN)

**Figure 1:**
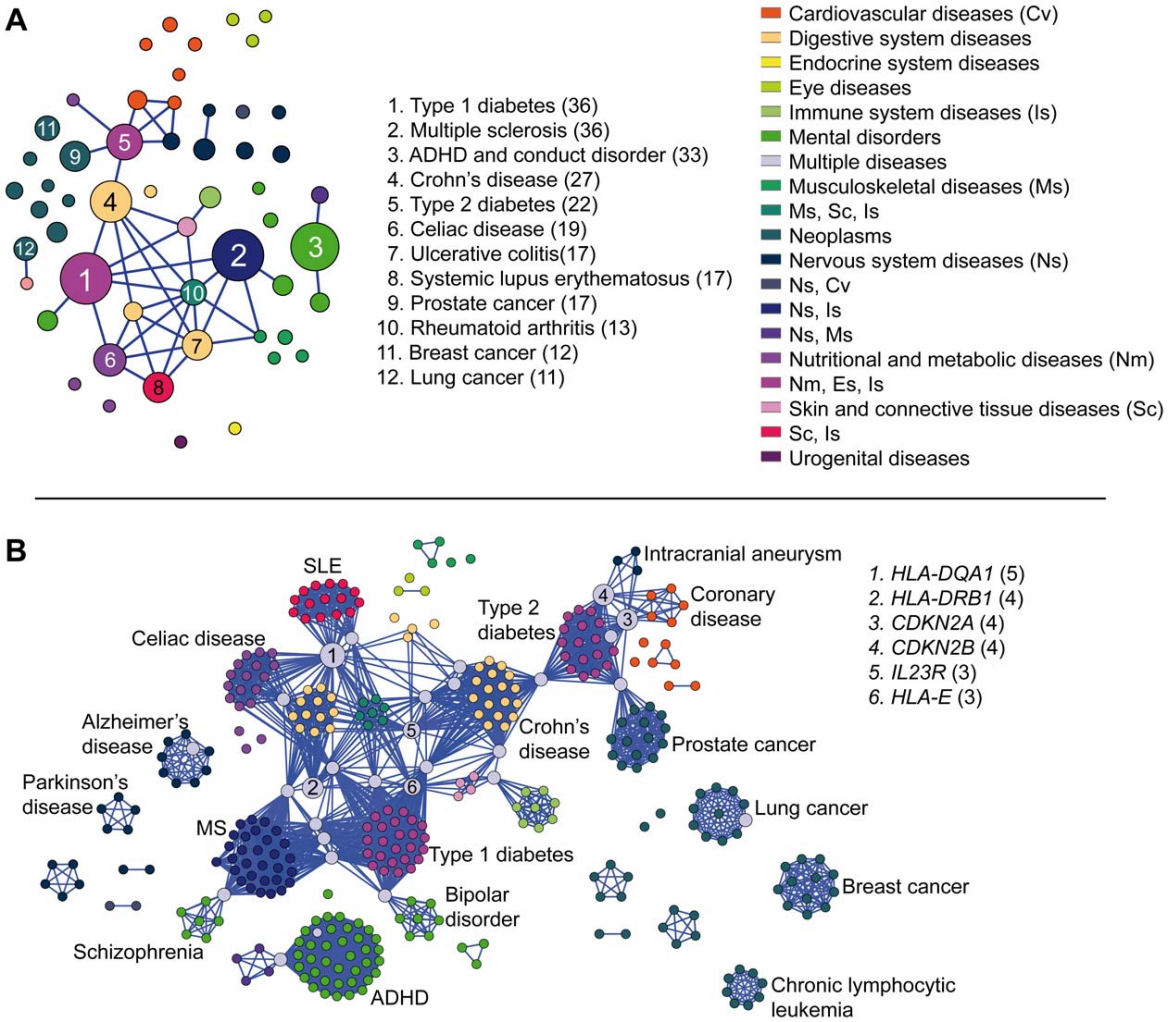
Networks of complex diseases and complex disease genes. **A**) Complex Disease Network (CDN). Each node is a complex disease studied in GWAs with the link representing sharing of disease genes. The color of the nodes corresponds to disease class as identified using MeSH (Medical Subject Headings) terms as given on the right side. Notably, complex diseases are hard to define using single MeSH term. The node size refers to the number of associated genes identified. Diseases with most number of associated genes identified through GWAs are listed on the right side with the numbers in the parenthesis indicating the number of associated genes. **B**) Complex Disease Gene Network (CGN): Each node represents a gene and connections between two genes represent their association with the same disease. The node size refers to the number of diseases a gene is associated with. Genes associated with many diseases are listed on the top right side with the number of diseases they are implicated with, in the parenthesis. A node (highlighted in gray) each in lung cancer and Alzheimer's disease gene cluster are singular associations in idiopathic pulmonary fibrosis and narcolepsy respectively.

Our complex disease network (CDN) consisted of 54 nodes and 41 edges. Most of the nodes were isolates with only 26 having at least one edge (Figure 1A). The node size in Fig. 1A corresponds to the number of genes associated with each disease. The differences in the node sizes could effectively represent the differences in the allelic architecture among these common complex diseases. Ideally, GWAs capture association of variants with a considerably higher minor allele frequency (MAF) and large effect sizes (referred hereafter as high-profile variants). This could mean that diseases like type 1 diabetes and multiple sclerosis (with 36 disease genes each) may involve a larger number of genes with high-profile variants than Parkinson's disease and restless legs syndrome that involve only 5 genes. It is tempting to speculate that the allelic architecture of diseases with fewer associated genes from GWA studies might have an overrepresentation of variants with relatively lesser MAF, or more modest allele effect sizes, or both. There were 17 diseases with only one associated gene, possibly questioning the role of high-profile variants in these diseases. Strikingly, attention deficit hyperactivity disorder and conduct disorder had 33 associated genes but belonged to an isolated cluster of only three nodes. This is a clear indication of different underlying pathophysiological mechanisms for these diseases compared to other diseases in our dataset. Furthermore, breast cancer (with 12 genes) and chronic lymphocytic leukemia (with 8 genes) did not have any edge. Notably, the number of studies performed and the sample size considered can also affect the number of genes identified and thus also our interpretations. An increase in the number of studies and the sample size might lead to the identification of new genes and hence expansion of CDN and CGN.

Most intriguingly, diseases perceived to share pathophysiological mechanisms (different forms of cancer, Type 1 and Type 2 diabetes, neurodegenerative diseases) were not directly connected in the CDN. In fact, the different cancer types did not show any overlapping genes. Moreover, even though type 1 and type 2 diabetes were both part of the same component, they were only linked indirectly with Crohn's disease acting as a connecting link. Links between mental and metabolic diseases in the CDN emphasize the emerging concept of convergent mechanisms underlying these classes of complex diseases [22]. The modular nature of human genetic diseases has generated a lot of enthusiasm of an easy way of predicting new disease genes for phenotypically similar diseases [23]. However, the CDN obtained from GWAs results did not provide evidence for modularity of phenotypically similar genetic diseases. This suggests that high-profile variants often constitute disjoint parts of the allelic spectra of phenotypically similar complex diseases.

### Complex Disease Gene Network (CGN)

The CGN consisted of 349 genes (nodes) and 3440 edges (Fig. 1B). In the CGN 349 nodes were connected to at least to one other gene and 214 belonged to the giant component. Very few genes were associated with multiple diseases (grey-colored in Fig. 1B). *HLA-DQA1*, *HLA-DRB1*, *CDKN2A*, *CDKN2B*, *IL23R* and *HLA-E* genes showed an association with more than two diseases. Most of the genes that were associated with more than one disease were inflammatory; exceptions being *CDKN2A* and *CDKN2B* that were involved in proliferation. Interestingly genes involved in cancers and certain nervous system diseases were rarely associated with any other diseases; genes associated with one type of cancer were not associated with other types. Given that the link between two genes represent their association to the same disease it would be interesting to evaluate the combined effect of their corresponding variants on the disease susceptibility. Contrary to the candidate-gene-based association studies, GWAs effectively overcome spurious associations, emphasizing that the genes associated with multiple diseases may possibly elevate the risk for all the diseases they are implicated with.

### Topological Properties of Complex Disease Genes in Human Interactome

We obtained PPIs from HPRD and constructed a molecular interaction network. One of the network measures we use to quantify this data with is the assortativity. This quantity measures the tendency for nodes with similar magnitude of degrees to be connected by an edge. This determines whether the nodes of large degrees are primarily linked to low-degree vertices or if low- and high-degree vertices are typically connected. Technically, the assortativity *r* is the Pearson correlation coefficient of degrees at either side of an edge, over the set of all edges. (Assortativity is further described in Supplementary Material S1.) We observed a negative assortativity of the human interactome; it was, however, larger than the randomized networks (–0.05 and –0.12 respectively; *P*<0.001). This means that, given the degrees of the interactome, it was actually wired with a bias towards degrees of similar magnitude being connected. Note, however, given that the range of possible *r*-values for a graph constrained to a specific set of degrees typically varies about 1.5 units [24], thus the magnitude of *r* for the real and rewired networks were close. So, even though the *P*-value indicated that the real *r* was significantly larger than our null-model, the indicated tendency could have been stronger, given the basic constraints of the null-model. The modularity (the tendency for the network to be divisible into dense regions that are sparsely interconnected, see Supplementary Material S1) was significantly larger than the randomized null model (0.53 and 0.32 respectively; *P*<0.001), which supported a modular organization of the PPI network. The overlap (see Supplementary Material S1) between annotated diseases and network clusters had a *z*-score of 3.9±0.1, meaning that the network clusters separated the disease genes so that genes belonging to the same category of disease were significantly more likely to belong to the same network cluster than expected by chance. The giant component of the human interactome consisted of 9045 nodes in comparison to 9281.6 nodes in the random network (*P*<0.001). This suggested that the human CGN shares topological similarities with that of the human interactome.

**Figure 2:**
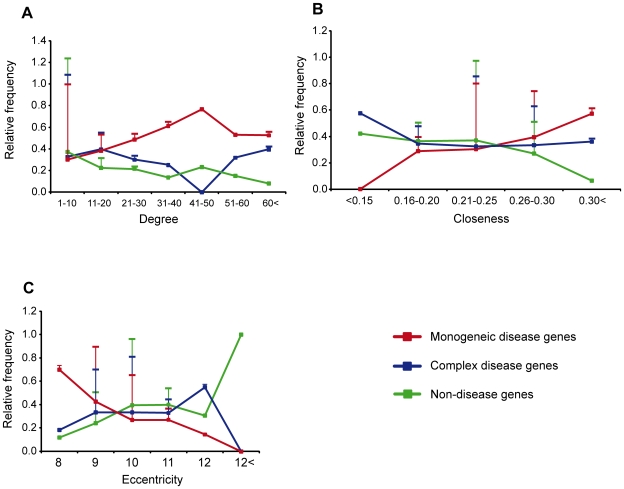
Comparative distribution of topological measures (A) Degree, (B) Closeness and (C). Eccentricity among monogenic disease genes, complex disease genes and non-disease genes in human interactome. The co-ordinates on the Y-axis indicate the relative frequency of each of the above mentioned classes in a given bin with the error bars indicating the fraction of genes in each bin for each class of genes. For example, monogenic disease genes are over-represented in the bin of 0.30< for closeness while closeness values for most of the genes in each class lie in the interval of 0.21–0.25.

We examined the topological properties of the complex disease genes in the human interactome by comparing them with that of monogenic disease genes and non-disease genes. We observed that the degrees of complex disease genes were significantly lower than those of monogenic disease genes (average degrees are 9.5 and 13.3 respectively; *P* = 6.4×10^−4^). Differences in closeness provided suggestive significance between these two groups (average values 0.24 and 0.25 respectively; *P* = 0.05) while complex disease genes had higher average eccentricity (9.8) compared to monogenic disease genes (9.6; *P* = 7.3×10^−4^). Comparisons with non-disease genes revealed that complex disease genes had higher degree, closeness and eccentricity (*P* = 6.3×10^−4^; 0.03 and 0.008 respectively). The respective average values for non-disease genes were 5.8, 0.23 and 9.9. The relative frequency of each class of genes in each interval of degree, closeness and eccentricity explicitly demonstrated this (Fig. 2 panels A, B and C respectively). From this we conclude that the complex disease genes are less central compared to the monogenic disease genes and occupy an intermediate niche between the monogenic disease genes and non-disease genes. However, knowledge bias associated with human interactome may affect the outcome of such comparisons. Higher network connectivity of essential genes has already been well documented [25]. Our results confirmed this observation, highlighting that essential genes have higher degree and closeness and lower eccentricity compared to complex disease genes (*P* = 2.6×10^−8^; 1.5×10^−9^ and 7.1×10^−4^ respectively). With the current understanding of the human interactome and the results presented here, the centrality of gene classes can be ordered as essential genes (being the most central), monogenic disease genes, complex disease genes and non-disease genes (being the most peripheral). Moving from the center to the periphery is thus moving from lethality, via disease to negligible effect of variations and thus reflects importance of functionality of the encoded proteins. For mutations in individual genes to be sufficient to manifest clinical phenotypes they should occur in less central regions of the interactome which do not affect the survival. That complex disease genes are relatively peripheral accentuates that variations in these genes are essential, but not sufficient, to result in a disease phenotype, which aid their maintenance and perpetuation in a population.

### Functional Properties of Complex Disease Genes in Human Interactome

Genes associated with the same disease tended to share a PPI more often than genes associated with different diseases (*P* = 5.1×10^−5^). Effectively, the average distance between genes causing the same disease was significantly lower than the average distance between genes associated with different diseases (*P* = 2.7×10^−5^). We observed a strong tendency for genes causing the same disease, compared to genes associated different diseases, to share the same GO term (13.3% and 8.9% respectively; *P* = 1.9×10^−12^). There seemed to be an overrepresentation of inflammatory genes among all the disease genes compared to all known genes, as can be seen in table 1. This might indicate that the common complex diseases pertinently involve inflammatory responses. Notably, such an observation may also result from the kind of diseases studied using GWAs so far.

**Table 1:** Overrepresented Gene Ontology (GO) biological processes among complex disease genes.

GO Term	Biological process description	*P*-value
GO:0002504	Response to molecule of bacterial origin	1.1×10^−6^
GO:0007050	Regulation of cytokine biosynthetic process	1.1×10^−6^
GO:0006955	Cellular calcium ion homeostasis	2.2×10^−6^
GO:0050715	Positive regulation of chemokine biosynthetic process	1.8×10^−5^
GO:0032800	Negative regulation of cytokine secretion during immune response	8.4×10^−5^
GO:0035095	Leukocyte differentiation	1.6×10^−4^
GO:0009967	Positive regulation of gene-specific transcription	2.9×10^−4^
GO:0032270	Behavioral response to nicotine	3.8×10^−4^
GO:0006954	Positive regulation of NK cell mediated toxicity directed against tumor cell target	3.8×10^−4^
GO:00032816	Antigen processing and presentation of peptide or polysaccharide antigen via MHC class II	4.2×10^−4^

## Discussion

In conclusion, here we have derived networks for complex diseases and complex disease genes based on GWAs, most of which have analyzed Caucasian population. With the information from GWAs done on other populations, we expect that CGN and CDN would expand. Nevertheless, GWAs mostly done on Caucasians adds strength to the analysis provided here, as these networks are derived from a homogenous population with isogenic background. The advantage of using GWAs data is that complex disease genes, unlike genes listed in OMIM, are identified without discovery bias. However, the results presented here are to be interpreted with caution as the genes considered here are identified to be associated with complex diseases and less is known about their role in disease causality. Other possible confounders include the kind of common complex diseases studied using GWAs and the knowledge-based bias associated with the human interactome. Notably, GWAs are empowered to track disease association of high-profile variants (higher MAF and/or high allele-effect size). So, the results presented here do not account for variants with modest allele effect size. The complex disease genes exhibited significantly different topological features compared to the monogenic disease genes. We, therefore, surmise that genes in the network neighborhood of complex disease genes should be prioritized in predicting new complex disease gene candidates. As the number of diseases studied using GWAs increases, improving the resolution of the CDN and CGN, we expect a better comprehension of the complex diseases, co-morbidities and the underlying mechanisms.

## Supporting Information

Supplementary Material S1 Description of topological network measures.
(0.70 MB PDF)

Table S1 Diseases analyzed using genome-wide association studies and their corresponding genes.
(0.10 MB PDF)
