# Supplementary material for: Network Properties of Complex Human Disease Genes Identified through Genome-Wide Association Studies
Source: PLoS One. 2009 Nov 30;4(11):e8090. doi: 10.1371/journal.pone.0008090 (PMC2779513; doi:10.1371/journal.pone.0008090)
Supplement: Supplementary Material S1 — Description of topological network measures. (0.70 MB PDF) [file pone.0008090.s001.pdf]

Network properties of complex human disease genes identified through genome-wide association studies

Fredrik Barrenas, Sreenivas Chavali, Petter Holme, Reza Mobini, Mikael Benson

Supplementary Material:

Network measures

In this Supplementary Material we will discuss the different network topological quantities that we use in a bit more technical detail than in the paper. In general we assume the network is represented as a graph $G = (V, E)$, where V is the set of vertices, or nodes, and E is the set of edges, or links, pairs of vertices. We assume G is a *simple graph*, i.e. that it does not have any loops (self-edges) or multiple edges.

Rewired graphs as null-models

Network structure [1,2] is how a network differs from a random network. It is, in other words, a relative concept, but network measures are usually not. To fully characterize network structure we need a random network to compare the measured quantities to. Thus, to measure network structure we need a reference- or null-model to compare the values for the real network to. The phrase "random networks" above is usually taken to mean random networks with the same fundamental constraints as the real system. The simplest null-model is the Erdős–Rényi model [3], where the sizes (number of nodes and edges) are fixed, everything else is random.

Following the works of Barabási *et al.* (summarized in Ref. [2]) the degree distribution has become accepted as the most fundamental network characteristic (apart from the sizes). With that starting point it has become a standard to compare network quantities to a null-model where the degrees of the network (the *degree sequence*) is fixed and everything else random. In modern network biology this method was first used in Refs. [4] and [5] but has a longer history than so [6] outside biology. On a philosophical note, using this model as a reference gives us information about the structure apart from what comes from the degrees. The degrees of gene networks are arguably mostly related to intrinsic factors of the physiochemical properties of transcription. However, one cannot rule out that intrinsic factors, how the network actually is wired, via evolution has influenced the degrees of the vertices. This observation leads us to believe that this null-model might not be the most appropriate for gene networks, but is probably

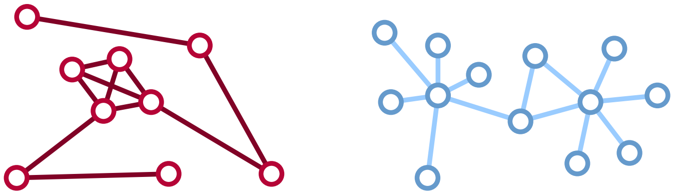

Fig. 1. An illustration of assortativity. The left figure shows graph with positive assortativity where large-degree nodes primarily attach to other large-degree nodes, and low degree nodes to low-degree nodes ($r = 0.36$). The right graph is disassortative, ($r = -0.91$), where the hubs connect to low-degree nodes

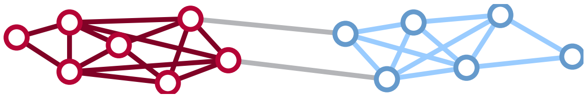

Fig. 2. Illustration of modularity. In this graph the modularity of the two partitions (red and blue). The modularity of this partition is 0.48 which is also the maximal modularity for this graph.

not far from it; and since it is commonly used, and developing a new null-model would be an ambitious research project in itself, we employ it.

The way to sample this null-model is straightforward — starting from the real network one chooses a pair of edges (*i,j*) and (*i',j'*) and, provided it does not introduce a self- or multiple edge, it is replaced by (*i,j'*) and (*i',j*). Throughout this procedure, all vertices keep their original degrees, but the information about how they were connected is lost. When every edge has been rewired at least once we have a sample of the null-model. Continuing the rewiring process until the all edges are rewired again gives us another instance of the null model. In our paper we repeat this process 1000 times to get 1000 samples of the null-model. The average values of the network measure over these samples are then the value we use for reference.

Assortativity

Is there a tendency of nodes with the same magnitude of degree to connect to each other, or are large-degree nodes primarily connected to low-degree nodes? This question is answered by the assortativity (or assortative mixing) coefficient r [7] — essentially the Pearson correlation coefficient of nodes at either side of an edge. The complication comes from the fact that Pearson's coefficient has a built in directionality — it measures the correlation between one variable as a function of another, whereas our question "one" is the same as "another". The solution is to consider undirected edges as two directed edges pointing in opposite directions. A practical formula for computer measurements of r is

$$r = (4\,\langle k_1\, k_2\rangle - \langle k_1 + k_2\rangle^2) \,/\, (2\,\langle k_1^{\,2} + k_2^{\,2}\rangle - \langle k_1 + k_2\rangle^2) \quad (1)$$

where $\langle \ldots \rangle$ denotes averages over all the edges, k_1 is the degree of the first argument of the edge in the internal representation of the edge, and k_2 is the degree of the second argument.

The assortativity ranges from −1 to +1, positive values meaning a tendency for nodes of similar degrees to connect to each other, negative values means that large-degree nodes tend to attach to low-degree nodes. One thing worth noting is that r is heavily influenced by the degree sequence of the graph [8]. Assuming the rewiring null-model described above, r of a completely random graph is negative. Zero represents neutrality of r with respect to a null-model of random multigraphs constrained only to the sizes (the number of vertices and edges). An illustration of assortativity can be seen in Fig. 1.

Centrality measures

While assortativity is a way to characterize the network as a whole, one can also use network measures to study individual nodes. One of the most fundamental network measures for nodes are those trying to capture the node's centrality [9]. There are several aspects on can take in assessing a nodes location between the center and the periphery. The simplest way (one can discuss if it really is a centrality measure) is the degree. Every node is at the center of its own neighborhood, a node high degree has a large neighborhood, and is thus at the center, myopically, of a relatively large part of the graph. The degree does not take the whole network into account. The simplest global centrality measure, or rather anti-centrality measure (the smaller the value is, the more central is the vertex), is eccentricity E

$$E = \max_j d(i,j) \,, \tag{2}$$

where $d(i,j)$ is the distance between i and j (number of edges in the shortest path between i and j). The eccentricity focus on a maximum of a property, and like maxima in general are sensitive for fluctuations (only one node needs to be added to a network to change the eccentricity by one unit). A more stable measure, that is arguable more relevant in for many biological processes, related to average than extremal performance, is the *closeness centrality*

$$C(i) = (N - 1) / \Sigma_{i \neq j} d(i,j) \tag{3}$$

Closeness centrality, the reciprocal average distances from one vertex to the rest of the graph focus on exactly what its name suggests. The node with highest closeness centrality is the node that, on average, can be reached by fewest steps in the graph from other vertices.

Network modularity

A network cluster is a region of the network that is strongly connected within and relatively sparsely connected to the rest of the network. Such clusters are interesting in biology because of their similarity to the notion of biological module — a relatively independent subsystem performing some biologically well-defined function. By this analogy we also call the clusters network modules, admitting the biological concept has a stronger focus on dynamic processes than its network counterpart. The common way of measuring how well a subdivision of a network into clusters capture the modular structure of the network is by the *network modularity* [10]

$$Q = \Sigma_i \, [e_{ii} - (\Sigma_j e_{ij})^2] \tag{4}$$

where e_{ij} is the fraction of the edges going between modules i and j. The first term gives a positive contribution to edges within a cluster; the second term penalizes edges between different clusters (the form is chosen so that the expected Q-value in a random multigraph is zero). A method to divide a graph into clusters is to find the cluster division that maximizes Q. This turns out to be a very hard computational problem and a large body of literature has been devoted to finding approximate solutions. We use the method proposed in Ref. 11.

Q, the maximal Q-value over all partitions of the graph, is a prototype measure of the modularity of a network. A clear cluster structure should give a large Q'-value. Just like assortativity gets a non-zero value for simple graph models, especially if they have broad degree distributions, so does Q' [12]. It is thus important to interpret Q' compared with a null-model.

Overlap between disease categories and network clusters

How are the disease genes clustered in the protein–protein interaction network? Are disease genes of one category spread out randomly between the network clusters, or do the network clusters and disease categories divide the network in the same way? To answer these questions this, we calculate an overlap score defined in Ref. 13. Let $\phi_{\Delta\Lambda}(\delta,\lambda)$ be the fraction of nodes associated with disease type δ (Δ is the set of diseases classes) in network cluster λ (Λ is the set of network clusters). In a network where the genes of the same type of diseases are grouped in the same network clusters $\phi_{\Delta\Lambda}$ will be either zero or relatively large, i.e. deviate much from its expected value, $\phi_\Delta(\delta)\, \phi_\Lambda(\lambda)$. From this observation we get the following overlap measure [13]

$$\nu = \Sigma_{\delta\in\Delta} \Sigma_{\lambda\in\Lambda} \left|\phi_{\Delta\Lambda}(\delta,\lambda) - \phi_\Delta(\delta)\, \phi_\Lambda(\lambda)\right| \tag{5}$$

that increases if diseases of the same type are increasingly often located to the same network clusters. In an infinite system, in random systems without the overlap we are interested in, v will be zero. In finite systems however, due to the absolute values, fluctuations will make the expectation value of v, even for systems with no correlation between network clusters and disease types, positive. To get around this problem we rather measure the z-score (deviation from mean divided by the standard deviation) with respect to a randomized reference model with the only constraint that the same disease type cannot be assigned the same node twice, and every node should belong to one and only one network cluster. For our data set of human genes and their annotated diseases we measure the z-score to 3.9 ± 0.1.

References

1. Newman MEJ. Structure and function of complex networks. SIAM Rev 2003; 45: 167–256.
2. Albert R, Barabási A-L. Statistical mechanics of complex networks. Rev Mod Phys 2002; 74: 47–92
3. Erdos P, Rényi A. On random graphs (I). Publ Math Debrecen 1959; 6: 290–7
4. Maslov S, Sneppen K. Specificity and stability in topology of protein networks. Science 2002; 296: 910–3
5. Shen-Orr S, Milo R, Mangan S, Alon U. Network motifs in the transcriptional regulation network of *Escherichia coli*. Nature Genetics 2002; 31: 64–8
6. Gale D. A theorem of flows in networks. Pacific J Math 1957; 7: 1073–82
7. Newman MEJ. Assortative mixing in networks. Phys Rev Lett 2002; 89: 208701
8. Holme P, Zhao J. Exploring the assortativity–clustering space of a network's degree sequence, Phys Rev E 2007; 75: 046111.
9. Buckley F, Harary F. Distances in graphs. Addison–Wesley, Redwood City, 1989.
10. Newman MEJ, Girvan M. Finding and evaluating community structure in networks. Phys Rev E 2004; 69: 026113.
11. Newman MEJ. Modularity and community structure in networks. Proc Natl Acad Sci USA 2006; 103: 8577–82
12. Guimerà R, Sales-Pardo M, Amaral LAN. Modularity from fluctuations in random graphs and complex networks. Phys Rev E 2003; 68: 065103
13. Holme P, Huss M. Substance networks are optimal simple-graph representations of metabolism. Under review with Bioinformatics.
