# Supplementary material for: Network Properties of Complex Human Disease Genes Identified through Genome-Wide Association Studies
Source: PLoS One. 2009 Nov 30;4(11):e8090. doi: 10.1371/journal.pone.0008090 (PMC2779513; doi:10.1371/journal.pone.0008090)
Supplement: Table S1 — Diseases analyzed using genome-wide association studies and their corresponding genes. (0.10 MB PDF) [file pone.0008090.s002.pdf]

Disease/Trait	Reported Gene	Entrez
ADHD and conduct disorder	A2BP1	54715
ADHD and conduct disorder	ABHD13	84945
ADHD and conduct disorder	AMOTL1	154810
ADHD and conduct disorder	ATP8B1	5205
ADHD and conduct disorder	C12orf28	196446
ADHD and conduct disorder	C16orf46	123775
ADHD and conduct disorder	C5orf15	56951
ADHD and conduct disorder	CWC15	51503
ADHD and conduct disorder	DHODH	1723
ADHD and conduct disorder	ETV3	2117
ADHD and conduct disorder	ETV3L	440695
ADHD and conduct disorder	FLJ31818	154743
ADHD and conduct disorder	FLJ39061	165057
ADHD and conduct disorder	FZD7	8324
ADHD and conduct disorder	GLT25D2	23127
ADHD and conduct disorder	GPR85	54329
ADHD and conduct disorder	GSX1	219409
ADHD and conduct disorder	JMJD2D	55693
ADHD and conduct disorder	KIAA0174	9798
ADHD and conduct disorder	KIRREL3	84623
ADHD and conduct disorder	LIG4	3981
ADHD and conduct disorder	LOC729257	729257
ADHD and conduct disorder	PAWR	5074
ADHD and conduct disorder	PDX1	3651
ADHD and conduct disorder	PITRM1	10531
ADHD and conduct disorder	PKD1L2	114780
ADHD and conduct disorder	PKD1L3	342372
ADHD and conduct disorder	PTPRD	5789
ADHD and conduct disorder	RGL1	23179
ADHD and conduct disorder	SPATA8	145946
ADHD and conduct disorder	YWHAZ	7534
Age-related macular degeneration	CFH	3075
AIDS progression	BAT1	7919
AIDS progression	HCP5	10866
AIDS progression	HLA-C	3107
AIDS progression	LTB	4050
AIDS progression	MCCD1	401250
AIDS progression	MICB	4277
AIDS progression	RNF39	80352
AIDS progression	TNF	7124
AIDS progression	ZNRD1	30834
Alzheimer's disease	APOC	341
Alzheimer's disease	APOE	348
Alzheimer's disease	CD33	945
Alzheimer's disease	CPT1B	1375
Alzheimer's disease	DISC1	27185
Alzheimer's disease	FAM113B	91523
Alzheimer's disease	PVRL2	5819
Alzheimer's disease	TOMM40	10452
Alzheimer's disease	ZNF224	7767

Amyotrophic lateral sclerosis	DPP6	1804
Amyotrophic lateral sclerosis	ITPR2	3709
Amyotrophic lateral sclerosis	KlAA1727	NA
Amyotrophic lateral sclerosis	LIPC	3990
Amyotrophic lateral sclerosis	SUSD1	64420
Amyotrophic lateral sclerosis	ZFP64	55734
ADHD symptoms (interaction)	KIF6	221458
ADHD symptoms (interaction)	PIWIL4	143689
Bipolar disorder	PALB2	79728
Bipolar disorder	ANK3	288
Bipolar disorder	C15orf53	400359
Bipolar disorder	CACNA1C	775
Bipolar disorder	DCTN5	84516
Bipolar disorder	DGKH	160851
Bipolar disorder	NDUFAB1	4706
Bipolar disorder	RASGRP1	10125
Breast cancer	TNRC9	27324
Breast cancer	ABCC4	10257
Breast cancer	COL1A1	1277
Breast cancer	ECHDC1	55862
Breast cancer	FBN1	2200
Breast cancer	FGFR2	2263
Breast cancer	GLG1	2734
Breast cancer	GRIK1	2897
Breast cancer	LOC643714	643714
Breast cancer	LSP1	4046
Breast cancer	MAP3K1	4214
Breast cancer	RNF146	81847
Celiac disease	TENR	132612
Celiac disease	ADAD1	132612
Celiac disease	ATXN2	6311
Celiac disease	CCR1	1230
Celiac disease	CCR3	1232
Celiac disease	HLA-DQA1	3117
Celiac disease	IL12A	3592
Celiac disease	IL18R1	8809
Celiac disease	IL18RAP	8807
Celiac disease	IL1RL1	9173
Celiac disease	IL2	3558
Celiac disease	IL21	50616
Celiac disease	KIAA1109	84162
Celiac disease	LPP	4026
Celiac disease	RGS1	5996
Celiac disease	SCHIP1	29970
Celiac disease	SH2B3	10019
Celiac disease	SLC9A4	389015
Celiac disease	TAGAP	117289
Chronic lymphocytic leukemia	STRN4	29888
Chronic lymphocytic leukemia	ACOXL	55289
Chronic lymphocytic leukemia	BCL2L11	10018
Chronic lymphocytic leukemia	GRAMD1B	57476

Chronic lymphocytic leukemia	IRF4	3662
Chronic lymphocytic leukemia	PRKD2	25865
Chronic lymphocytic leukemia	SP110	3431
Chronic lymphocytic leukemia	SP140	11262
Colorectal cancer	BMP4	652
Colorectal cancer	CDH1	999
Colorectal cancer	DQ515897	NA
Colorectal cancer	EIF3H	8667
Colorectal cancer	HsG57825	NA
Colorectal cancer	ORF DQ515897	NA
Colorectal cancer	POU5FIP1	NA
Colorectal cancer	RHPN2	85415
Colorectal cancer	SMAD7	4092
Conduct disorder (interaction)	A2BP1	54715
Conduct disorder (interaction)	ADH1C	124
Conduct disorder (interaction)	MFHAS1	9258
Conduct disorder (interaction)	PPM1K	152926
Conduct disorder (interaction)	RIT1	6016
Conduct disorder (interaction)	SLC6A1	6529
Conduct disorder (interaction)	ZBTB16	7704
Coronary artery calcification	NUMB	8650
Coronary artery calcification	DR1M	NA
Coronary disease	CDKN2A	1029
Coronary disease	CDKN2B	1030
Coronary disease	CXCL12	6387
Coronary disease	MIA3	375056
Coronary disease	MTHFD1L	25902
Coronary disease	PSRC1	84722
Coronary disease	SMAD3	4088
Creutzfeldt-Jakob disease	STMN2	11075
Creutzfeldt-Jakob disease	PRNP	5621
Crohn's disease	ATG16L1	55054
Crohn's disease	BSN	8927
Crohn's disease	C11orf30	56946
Crohn's disease	CARD15	64127
Crohn's disease	CCR6	1235
Crohn's disease	CDKAL1	54901
Crohn's disease	ICOSLG	23308
Crohn's disease	IL12B	3593
Crohn's disease	IL23R	149233
Crohn's disease	IRGM	345611
Crohn's disease	ITLN1	55600
Crohn's disease	JAK2	3717
Crohn's disease	LRRK2	120892
Crohn's disease	MST1	4485
Crohn's disease	MUC19	283463
Crohn's disease	NKX2-3	159296
Crohn's disease	NOD2	64127
Crohn's disease	ORMDL3	94103
Crohn's disease	PTGER4	5734
Crohn's disease	PTPN2	5771

Crohn's disease	PTPN22	26191
Crohn's disease	STAT3	6774
Crohn's disease	TNFSF15	9966
Crohn's disease	ZNF365	22891
Crohn's disease and Sarcoidosis (combined)	C10orf67	256815
Cutaneous basal cell carcinoma	RHOU	58480
Cutaneous basal cell carcinoma	ARHGEF10L	55160
Cutaneous basal cell carcinoma	PADI4	23569
Cutaneous basal cell carcinoma	PADI6	353238
Cutaneous basal cell carcinoma	RCC2	55920
Diabetes related insulin traits	CPVL	54504
Diabetic nephropathy	ELMO1	9844
Early onset extreme obesity	FTO	79068
End-stage renal disease	PVT1	5820
Exfoliation glaucoma	LOXL1	4016
Gallstones	ABCG8	64241
Heart failure	KIAA1598	57698
Hypertension	ZP4	57829
Hypertension	RYR2	6262
Hypertension	CHRM3	1131
Idiopathic pulmonary fibrosis	TERT	7015
Incident diabetes	TMEFF2	23671
Inflammatory bowel disease	CARD15	64127
Inflammatory bowel disease	HLA-DQA1	3117
Inflammatory bowel disease	IL23R	149233
Inflammatory bowel disease	NOD2	64127
Inflammatory bowel disease	PSMG1	8624
Inflammatory bowel disease	TNFRSF6B	8771
Inflammatory bowel disease	TNFSF15	9966
Inflammatory bowel syndrome	PTGER4	5734
Inflammatory bowel syndrome	NELL1	4745
Intracranial aneurysm	BOLL	66037
Intracranial aneurysm	CDKN2A	1029
Intracranial aneurysm	CDKN2B	1030
Intracranial aneurysm	PLCL1	5334
Intracranial aneurysm	SOX17	64321
Juvenile idiopathic arthritis	HLA-DRB1	3123
Kawasaki disease	ZFHX3	463
Kawasaki disease	NAALADL2	254827
Knee osteoarthritis	PTGS2	5743
Knee osteoarthritis	PARD3B	117583
Knee osteoarthritis	PLA2G4A	5321
Lung cancer	BAT3	7917
Lung cancer	CHRNA3	1136
Lung cancer	CHRNA5	1138
Lung cancer	CHRNB4	1143
Lung cancer	CLPTM1	1209
Lung cancer	CRP	1401
Lung cancer	IL1RAP	3556
Lung cancer	LOC123688	123688
Lung cancer	MSH5	4439

Lung cancer	PSMA4	5685
Lung cancer	TERT	7015
Major depressive disorder	CCND2	894
Melanoma	CDC91L1	128869
Multiple sclerosis	C20orf46	55321
Multiple sclerosis	CSMD1	64478
Multiple sclerosis	DBC1	1620
Multiple sclerosis	DDEF2	8853
Multiple sclerosis	EN1	2019
Multiple sclerosis	GPC5	2262
Multiple sclerosis	HLA-DRA	3122
Multiple sclerosis	HLA-DRB1	3123
Multiple sclerosis	IL2RA	3559
Multiple sclerosis	IL7RA	3575
Multiple sclerosis	KIAA0350	23274
Multiple sclerosis	KIF1B	23095
Multiple sclerosis	MGC45800	90768
Multiple sclerosis	PDZRN4	29951
Multiple sclerosis	RPL5	6125
Multiple sclerosis	SH3GL2	6456
Multiple sclerosis	SLC25A36	55186
Multiple sclerosis	ZIC1	7545
Multiple sclerosis (age of onset)	C1orf125	126859
Multiple sclerosis (age of onset)	FLJ34870	344148
Multiple sclerosis (age of onset)	KCNB2	9312
Multiple sclerosis (age of onset)	RAB38	23682
Multiple sclerosis (age of onset)	RELN	5649
Multiple sclerosis (age of onset)	RFK	55312
Multiple sclerosis (age of onset)	SGCD	6444
Multiple sclerosis (age of onset)	WDR7	23335
Multiple sclerosis (severity)	ACP5	54
Multiple sclerosis (severity)	C16orf47	388289
Multiple sclerosis (severity)	C1GALT1	56913
Multiple sclerosis (severity)	CBLN2	147381
Multiple sclerosis (severity)	CENPC1	1060
Multiple sclerosis (severity)	FLJ16641	389170
Multiple sclerosis (severity)	JARID2	3720
Multiple sclerosis (severity)	LOC132321	NA
Multiple sclerosis (severity)	MET	4233
Multiple sclerosis (severity)	MGC13125	84811
Multiple sclerosis (severity)	NLRP11	204801
Myocardial infarction	CDKN2B	1030
Myocardial infarction	CDKN2A	1029
Myopathy	SLCO1B1	10599
Narcolepsy	CPT1B	1375
Neuroblastoma	FLJ22536	401237
Neuroblastoma	FLJ44180	NA
Neuroticism	AK127771	NA
Neuroticism	MAMDC1	161357
Neuroticism	NXPH1	30010
Neuroticism	PDE4D	5144

Osteonecrosis of the jaw	CYP2C8	1558
Parkinson disease (familial)	GAK	2580
Parkinson disease (familial)	DGKQ	1609
Parkinson's disease	BRDG1	26228
Parkinson's disease	DLG2	1740
Parkinson's disease	SEMA5A	9037
Prostate cancer	CTBP2	1488
Prostate cancer	CTDSPL	10217
Prostate cancer	EHBP1	23301
Prostate cancer	GSPT2	23708
Prostate cancer	HAPLN1	1404
Prostate cancer	HNF1B	6928
Prostate cancer	JAZF1	221895
Prostate cancer	KLK3	354
Prostate cancer	LMTK2	22853
Prostate cancer	LOC340602	340602
Prostate cancer	MAGED1	9500
Prostate cancer	MSMB	4477
Prostate cancer	NUDT10	170685
Prostate cancer	NUDT11	55190
Prostate cancer	PKHD1	5314
Prostate cancer	SLC22A3	6581
Prostate cancer	TCF2	6928
Psoriasis	COG6	57511
Psoriasis	HLA-C	3107
Psoriasis	IL12B	3593
Psoriasis	LCE3A	353142
Psoriasis	LCE3D	84648
Psoriasis	MHC	3133
Psoriasis	SPATA2	9825
Restless legs syndrome	BTBD9	114781
Restless legs syndrome	LBXCOR1	390598
Restless legs syndrome	MAP2K5	5607
Restless legs syndrome	MEIS1	4211
Restless legs syndrome	PTPRD	5789
Rheumatoid arthritis	CCL21	6366
Rheumatoid arthritis	CD40	958
Rheumatoid arthritis	CDK6	1021
Rheumatoid arthritis	HLA-DQA1	3117
Rheumatoid arthritis	HLA-DQA2	3118
Rheumatoid arthritis	HLA-DRB1	3123
Rheumatoid arthritis	MHC	3133
Rheumatoid arthritis	OLIG3	167826
Rheumatoid arthritis	PRKCQ	5588
Rheumatoid arthritis	PTPN22	26191
Rheumatoid arthritis	SALL3	27164
Rheumatoid arthritis	TNFAIP3	7128
Rheumatoid arthritis	TNFIP3	21929
Rheumatoid arthritis	TRAF1-C5	NA
Schizophrenia	ZNF804A	91752
Schizophrenia	RELN	5649

Schizophrenia	ACSM1	116285
Schizophrenia	AGBL1	123624
Schizophrenia	BUCS1	116285
Schizophrenia	CCDC60	160777
Schizophrenia	CSF2RA	1438
Schizophrenia	IL3RA	3563
Stroke	IMPA2	3613
Stroke	AIM1	202
Systemic lupus erythematosus	BANK1	55024
Systemic lupus erythematosus	BLK	640
Systemic lupus erythematosus	c10orf64	57705
Systemic lupus erythematosus	C8orf13	83648
Systemic lupus erythematosus	GHR	2690
Systemic lupus erythematosus	HLA-DQA1	3117
Systemic lupus erythematosus	IRF5	3663
Systemic lupus erythematosus	ITGAM	3684
Systemic lupus erythematosus	ITGAX	3687
Systemic lupus erythematosus	NEGR1	257194
Systemic lupus erythematosus	NTNG2	84628
Systemic lupus erythematosus	SOCS6	9306
Systemic lupus erythematosus	STAT4	6775
Systemic lupus erythematosus	TNFAIP3	7128
Systemic lupus erythematosus	TNPO3	23534
Systemic lupus erythematosus in women	KIAA1542	57661
Systemic lupus erythematosus in women	PXK	54899
Type 1 diabetes	UBASH3A	53347
Type 1 diabetes	AFF3	3899
Type 1 diabetes	BACH2	60468
Type 1 diabetes	C12orf30	80018
Type 1 diabetes	C16orf75	116028
Type 1 diabetes	C1QTNF6	114904
Type 1 diabetes	CAPSL	133690
Type 1 diabetes	CD226	10666
Type 1 diabetes	CDK2	1017
Type 1 diabetes	CLEC16A	23274
Type 1 diabetes	CTLA4	1493
Type 1 diabetes	CTSH	1512
Type 1 diabetes	EDG7	23566
Type 1 diabetes	ERBB3	2065
Type 1 diabetes	GLIS3	169792
Type 1 diabetes	HLA	NA
Type 1 diabetes	HLA-E	3133
Type 1 diabetes	IFIH1	64135
Type 1 diabetes	IKZF4	64375
Type 1 diabetes	IL2RA	3559
Type 1 diabetes	IL7R	3575
Type 1 diabetes	INS	3630
Type 1 diabetes	KIAA0350	23274
Type 1 diabetes	LNK	10019
Type 1 diabetes	LOC150577	150577
Type 1 diabetes	MHC	3133

Type 1 diabetes	PHTF1	10745
Type 1 diabetes	PRKCQ	5588
Type 1 diabetes	PRM3	58531
Type 1 diabetes	PTPN1	5770
Type 1 diabetes	PTPN2	5771
Type 1 diabetes	RAB5B	5869
Type 1 diabetes	RASGRP1	10125
Type 1 diabetes	SH2B3	10019
Type 1 diabetes	SUOX	6821
Type 1 diabetes	TNP2	7142
Type 1 diabetes	TRAFD1	10906
Type 2 diabetes	ADAM30	11085
Type 2 diabetes	ADAMTS9	56999
Type 2 diabetes	CAMK1D	57118
Type 2 diabetes	CDC123	8872
Type 2 diabetes	CDKAL	NA
Type 2 diabetes	CDKAL1	54901
Type 2 diabetes	CDKN2A	1029
Type 2 diabetes	CDKN2B	1030
Type 2 diabetes	DCD	117159
Type 2 diabetes	FTO	79068
Type 2 diabetes	HHEX	3087
Type 2 diabetes	IGF2BP2	10644
Type 2 diabetes	JAZF1	221895
Type 2 diabetes	KCNJ11	3767
Type 2 diabetes	LGR5	8549
Type 2 diabetes	NOTCH2	4853
Type 2 diabetes	PPARG	5468
Type 2 diabetes	SLC30A8	169026
Type 2 diabetes	SYN2	6854
Type 2 diabetes	TCF7L2	6934
Type 2 diabetes	THADA	63892
Type 2 diabetes	TSPAN8	7103
Type 2 diabetes	VEGFA	7422
Ulcerative colitis	BTNL2	56244
Ulcerative colitis	DLD	1738
Ulcerative colitis	HLA-DQA1	3117
Ulcerative colitis	HLA-DQB1	3119
Ulcerative colitis	HLA-DRA	3122
Ulcerative colitis	HLA-DRB1	3123
Ulcerative colitis	HLA-DRB5	3127
Ulcerative colitis	IFNG	3458
Ulcerative colitis	IL10	3586
Ulcerative colitis	IL22	50616
Ulcerative colitis	IL23R	149233
Ulcerative colitis	IL26	55801
Ulcerative colitis	LAMB1	3912
Ulcerative colitis	OTUD3	23252
Ulcerative colitis	PLA2G2E	30814
Ulcerative colitis	RNF186	54546
Ulcerative colitis	SLC26A3	1811

Urinary bladder cancer	TP63	8626
Urinary bladder cancer	MYC	4609
Urinary bladder cancer	BC042052	NA
Wet age-related macular degeneration	HTRA1	5654
